# Radiomics Approaches for the Prediction of Pathological Complete Response after Neoadjuvant Treatment in Locally Advanced Rectal Cancer: Ready for Prime Time?

**DOI:** 10.3390/cancers15020432

**Published:** 2023-01-09

**Authors:** Vincent Bourbonne, Ulrike Schick, Olivier Pradier, Dimitris Visvikis, Jean-Philippe Metges, Bogdan Badic

**Affiliations:** 1 Radiation Oncology Department, University Hospital, 29200 Brest, France; 2 LaTIM, INSERM, UMR 1101, University of Western Brittany, 29238 Brest, France; 3 Medical Oncology Department, University Hospital, 29200 Brest, France; 4 Digestive Surgery Department, University Hospital, 29200 Brest, France

**Keywords:** locally advanced rectal cancer, complete response, prediction, radiomics, watch-and-wait

## Abstract

**Simple Summary:**

Therapeutic management of locally advanced rectal cancer has seen profound modifications in the latest years, mainly with the advent of total neoadjuvant therapy. Adding neoadjuvant chemotherapy to neoadjuvant chemoradiotherapy leads to an improvement in locoregional disease-free survival. Organ preservation rises as an option for patients with complete response after neoadjuvant therapy without compromising overall survival. A current challenge that remains is the ever-growing need for selection tools to enable a personalized therapeutic approach for each patient. In this article, we review the place of different biomarkers (clinical, biological, genomics, transcriptomics, proteomics, and radiomics) as well as their clinical implementation and discuss the most recent trends for future steps in prediction modeling in patients with locally advanced rectal cancer.

**Abstract:**

In recent years, neoadjuvant therapy of locally advanced rectal cancer has seen tremendous modifications. Adding neoadjuvant chemotherapy before or after chemoradiotherapy significantly increases loco-regional disease-free survival, negative surgical margin rates, and complete response rates. The higher complete rate is particularly clinically meaningful given the possibility of organ preservation in this specific sub-population, without compromising overall survival. However, all locally advanced rectal cancer most likely does not benefit from total neoadjuvant therapy (TNT), but experiences higher toxicity rates. Diagnosis of complete response after neoadjuvant therapy is a real challenge, with a risk of false negatives and possible under-treatment. These new therapeutic approaches thus raise the need for better selection tools, enabling a personalized therapeutic approach for each patient. These tools mostly focus on the prediction of the pathological complete response given the clinical impact. In this article, we review the place of different biomarkers (clinical, biological, genomics, transcriptomics, proteomics, and radiomics) as well as their clinical implementation and discuss the most recent trends for future steps in prediction modeling in patients with locally advanced rectal cancer.

## 1. Introduction

Colorectal cancer is the third most frequent cancer, with approximately two million cases per year, and it represents the second leading cause of death by cancer [1]. Rectal cancer accounts for a third of all colorectal cancers. Therapeutic management of rectal cancer is based on the tumor level as well as the tumor staging. Locally advanced rectal cancer (LARC) is defined by a T3-T4 tumor and/or a nodal involvement, with no metastatic sites. In recent years, therapeutic management of LARCs has seen tremendous modifications, especially with the advent of total neoadjuvant therapy (TNT) and non-operative management (NOM). Adding neoadjuvant chemoradiotherapy (CRT) with a total dose of 50–56 Gy to the tumor before surgery was associated with increased loco-regional disease-free survival (LRDFS), higher pathological complete response rates (pCR), and higher negative surgical margin rates [2,3,4,5]. Neoadjuvant CRT did not improve metastasis-free survival (MFS) or overall survival (OS). Short course radiotherapy (SCRT) delivered in 25 Gy/5 fractions over a week is also associated with lower local recurrences [6,7,8]. Neoadjuvant CRT alone was thus the standard of care for all LARCs before being challenged by TNT. Indeed, the addition of neoadjuvant chemotherapy to neoadjuvant CRT was associated with improved LRDFS and pCR rates [9,10]. TNT did not impact negative surgical margins rates or OS [11,12,13,14].

For all LARCs, surgery has long been the cornerstone. Reaching pCR rates as high as 25% in group B of the CARO/ARO/AIO-12 trial [15] (consolidative CT after CRT) and 28% in the RAPIDO and PRODIGE-23 trials, total neoadjuvant therapy, compared to 12–14% rates with neoadjuvant CRT alone, is particularly interesting in the management of LARCs. Rates of 40–60% were even described. In patients presenting with pCR, careful monitoring and NOM was safe, with no impact on survival endpoints, and nearly half of the patients did not undergo surgery. Interestingly, if proposed, 83% of patients would opt for a NOM strategy [16] even if warned of potential worse outcomes in the case of local regrowth, as further discussed.

These pCR improvements come with the price of nonnegligible toxicity rates. In the PRODIGE-23 trial, for instance, grade 3–4 adverse events (AEs) occurred in 46% of patients during the neoadjuvant chemotherapy (CT) phase. Selection of patients is thus of paramount importance, with several treatment choices to be made between CRT alone, TNT with short or long CRT, induction or consolidative CT, and total mesorectal excision or NOM.

Assessment and prediction of pCR as well as other clinically meaningful endpoints have been extensively researched, with most studies focusing on the prediction of pCR. Despite the interesting results, none of the models are recommended in current guidelines. In this review, we review the place of different biomarkers (clinical, biological, genomics, transcriptomics, proteomics, and radiomics) as well as their clinical implementation and discuss the most recent trends for future steps in prediction modeling in patients with locally advanced rectal cancer.

## 2. Role of Clinical and Pathological Complete Response

Survival outcomes are significantly correlated with response to neoadjuvant CRT. In a cohort of 1089 patients [17], pCR patients harbored a significantly longer 5-year overall survival, 5-year disease-free survival, and 5-year distant recurrence-free survival. Pooled together, patients with a pCR and a near pCR response experienced an increased 5-year local recurrence-free survival.

Pooling data from 27 articles (17 different datasets), pCR was found in 484/3105 included patients (15.6%) [18]. Five-year disease-free survival was found in 83.3% (78.8–87.0%) of patients with pCR compared to 65.6% (63.6–68.0%) of non-pCR patients, resulting in an adjusted HR of 0.45. The role of pCR was also confirmed for local recurrence-free survival (HR 0.33 CI95% 0.19–0.60), distant metastasis-free survival (HR 0.40 CI95% 0.29–0.55), and overall survival (HR 0.51 CI95% 0.38–0.67). Of note, 8/17 studies were retrospective cohort studies. None were in the TNT setting.

The positive impact of pCR on survival outcomes leads to the development of non-surgical management, the main challenge then being the correlation between clinical complete response and pathological complete response.

Digital rectal examination (DRE), endoscopy, and magnetic resonance imaging (MRI) are the foundation for the local evaluation of tumor response following neoadjuvant therapy (NAT). The combined accuracy of all three modalities in predicting tumor absence is reported to be 98% [19]. A smooth, normal mucosa should be seen on DRE, though some slight abnormalities or a soft scar may be palpable. A flat white scar, telangiectasia, and the lack of an ulcer or nodule are the endoscopic features of cCR. In addition, there are no apparent lymph nodes or restricted diffusion [20].

One of the first reports of the watch-and-wait approach was made in 2011 by Maas et al. Within a cohort of 21 patients and a mean follow-up of 25 +/− 19 months, only one patient developed a local recurrence [21]. Similar reports were later published confirming the feasibility of the NOM. In a phase II randomized trial, 324 patients were compared with regard to the treatment sequence as well as survival endpoints with regard to the treatment strategy (watch-and-wait or surgery). No differences were found between groups in local recurrence-free survival, distant metastasis-free survival, and overall survival [22].

In a retrospective cohort of 113 patients, 22 patients experienced a local regrowth and were treated by salvage surgery. To be noted, a worse overall survival along with worse distant metastasis-free survival was noted in patients with local regrowth vs. those without local regrowth [23], confirming the absolute need for robust pCR prediction models to better personalize the treatments. Given the low rate of pCR, each model should be developed aiming for the highest balance between negative predictive value (NPV) and positive predictive value (PPV). The model should at first limit the risk of local relapse before opening the possibility of NOM. Each model should also be cost-effective, reproducible, and easily accessible. Given the place of MRI and other medical imaging modalities before CRT and the absence of additional costs, the radiomics model will be treated separately.

## 3. Prediction of pCR Using Non-Radiomics Model

Several approaches were proposed for the prediction of pCR and near-pathological complete response (nCR). In a systematic review summarizing 85 studies [24], several predictors (clinicopathological features, radiomics, gene expression, mutational and protein expression analyses) were associated with pCR. However, none appeared as a clear and robust biomarker. Indeed, only a fraction of the proposed models have been externally validated, and none have been incorporated into international guidelines. The advent of TNT, the development of NOM, and the recent advances in prediction modeling call for a new overview on this matter.

### 3.1. Clinical Predictors and Models

The vast majority of the published articles are based on either retrospective cohorts or secondary analysis of prospective trials. Prediction of pCR is often the main endpoint, clinical complete response (cCR) being used as a surrogate.

Assessment of cCR relies on DRE, post-CRT MRI, and endoscopy. In a recent meta-analysis, sensitivities/specificities of MRI, endorectal ultrasonography, and computed tomography (CT) were as high as 95%/31%, 97%/30%, and 96%/21%, respectively [25].

The role of endoscopy and especially post-CRT biopsy for the prediction of pCR was evaluated in a cohort of 198 patients, among which 186 underwent surgery [26]. Two features appeared as strong predictors of pCR: flattening of marginal swelling and negative post-CRT biopsies. Cancer-negative biopsy achieved the highest specificity (along with a low carcinoembryonic antigen), reaching 78.3%. However, sensitivity was the second lowest (65.0%). Consecutively, residual cancer was found in 76.0% of cases negative for cancer by endoscopic biopsy, proving the difficulty of pCR assessment.

Several clinical features are commonly accepted to increase the likelihood of pCR: low carcinoembryonic antigen (CEA) [27,28,29,30], small tumor size [31,32], low tumor/nodal stage [33], low histologic grade [33], small circumferential tumor extent [34], high hemoglobin levels [27,34], and a low neutrophil-to-lymphocyte ratio [35].

Using a propensity-matched cohort of 322 patients (161 in each group: cCR vs. no cCR), Mbanu et al. identified pre-treatment tumor diameter, tumor stage on magnetic resonance imaging (MRI), hemoglobin, alkaline phosphate, total radiotherapy depths, neutrophil-to-lymphocyte ratio (NLR), neutrophil-monocyte-to-lymphocyte ratio (NMLR), lymphocyte count, and albumin level as significant predictors of cCR [36]. Combining these parameters led to a nomogram with an area under the curve (AUC) of 0.75. Though the correlation between the fitted probability of cCR and observed cCR appeared to be interesting, no probability threshold was proposed.

In a wider cohort of 514 patients treated between 2004 and 2019 with mainly three-dimensional (3D) RT and two-dimensional (2D) RT with a total dose of 45 Gy to the whole pelvis and a tumor boost, longitudinal tumor diameter, extramural tumor invasion depth, CEA, hemoglobin levels, age, and interval between CRT and surgery were combined in a nomogram. A ROC of 0.72 (95%CI 0.68–0.77) was obtained for the prediction of good response defined by a Dworak tumor regression grade of 3 or 4 [37].

Among the identified clinical predictors, the delay between chemoradiotherapy and surgery appears to be the only one to be modifiable. The only prospective randomized trial to address the matter of timing remains the Lyon R90-01, in which pCR rates were analyzed with a 6–8-week interval compared with a shorter interval of 2 weeks [38]. To our knowledge, such a study was not conducted on the TNT era.

In one of the largest single-institution cohorts to date (1089 patients), 198 patients (18.2%) experienced a pCR [17]. No significant differences between the pCR and non-pCR patients were found regarding age, sex, vascular invasion, or CEA level. Tumor size, clinical node negativity, and differentiation were the only significant predictors. Of note, the delay between CRT and surgery was not available.

Despite the relative heterogeneity between these studies, several clinical features stood out. Tumor stage, the interval between CRT and surgery, nodal stage, histological differentiation, as well as biological parameters (CEA and hemoglobin) appeared to be consensual between most studies. Post-CRT negative biopsies reached a high specificity but remained more invasive.

### 3.2. Role of MRI and PET/CT

The size of the tumor and the nodes as well as tumor regression grade defined by qualitative assessment of change in signal intensity based on MRI are routinely used for pre-CRT tumor staging and for the assessment of response to therapy [39,40,41]. Where pre-CRT tumor volume does not seem effective for the prediction of pCR in most studies, post-CRT tumor evaluation was confirmed in several studies [31,42,43,44,45,46]. In a cohort of 64 patients, Sathyakumar et al. identified a complete response (ydwiT0) on post-CRT diffusion-weighted imaging (DWI) tumor volume with a reduction rate of >95% as the best predictor of pCR, with AUC values of 0.88 (CI 95% 0.74–1) and 0.84 (CI 95% 0.7–0.98), respectively [47]. Agreement for ydwiT0 was scored as moderate to high among three observers, with kappa values ranging from 0.79 to 0.90. The value of tumor shrinkage was also confirmed by other teams [44,48], highlighting the value of pre-/post-CRT evaluation and comparison. Regarding the choice of sequences to focus on, though the T2 morphological sequence has the advantage of availability and reproducibility, DWI and apparent diffusion coefficient (ADC) maps could help in differentiating residual tumor from fibrosis, but with conflicting results depending on the studies [49,50,51,52,53,54]. Similar results were obtained regarding dynamic contrast-enhanced imaging (DCE) [55,56].

MRI longitudinal monitoring in the context of MR-guided RT could also help in the stratification of patients between fast and slow regressors, but with no significant impact on 2-year DFS [57]. The distribution of response rates was not affected by the regression type, with only one complete response rate on an overall cohort of 20 patients limiting the power of the study. Similarly, in a cohort of 43 patients, MRI was conducted before, during, and after CRT. Variations in T2 and Diffusion B1000 values were predictors of response to treatment [58].

In a systematic review published in 2014, Memon et al. identified 17 series assessing PET prediction of pCR [59]. In cohorts varying from 20 to 151 patients, the most reliable predictors appeared to be a maximum standard uptake value at the time of post-CRT evaluation (SUV2Max), reduction in SUVMax (RISUVMax), and visual complete metabolic response. Of note, though a metabolic complete response was achieved for 23–60% of the patients, the pCR rate ranged from 6 to 35%.

### 3.3. Genomics, Transcriptomics

Based on a cohort of 38 patients, Gonçalves-Ribeiro et al. developed a two-protein immunohistochemical score that was further validated on an external cohort of 36 patients. Combining FN1 and COL3A1 with a logistic regression approach, the model was significantly associated with the tumor regression grade and pathological nodal stage and a statistical trend towards pCR [60]. Interestingly, only very few differences were observed between responders and non-responders with regard to tumor cells themselves, whereas the stroma displayed a profound transcriptomic change, as previously suggested [61,62]. The value of peri-tumoral tissues, notably in a radiomics workflow, should be further studied.

In a prospective cohort of 80 patients treated with CRT and subsequent surgery, four biomarkers (p53, p21, Ki67, and CD133) were identified as significantly correlated with TRG and pCR. A model based on the value of these four biomarkers significantly stratified patients according to their chance of pCR (14.5% vs. 83.3%, *p* < 0.001), with a positive predictive value (PPV) of 83.3% and a NPV of 85.5%. No test or validation sets were available [63]. The value of gene expression levels for the prediction of pCR was previously explored with different biomarkers being identified (LRR1Q3, FRMD3, SAMD5, and TMC7), but on smaller cohorts with, again, no validation sets [64].

The value of microsatellite instability (MSI) to predict pCR remains controversial. In a recent meta-analysis of 5086 patients (636 MSI-positive), MSI-positive tumors were associated with a reduction in pCR [65], whereas in a second meta-analysis, MSI status had no impact on pCR rate [66]. Similarly, due to low-volume cohorts, the role of the EGFR, MAPK/AKT, and pi3K/AKT/mTOR pathways should be further explored, especially given the recent development of specific pathway inhibitors such as ipatasertib [67].

### 3.4. Circulating Tumor Cells, Tissue, and Circulating miRNAs

There is great interest in pre- and post-treatment circulating DNA (ctDNA) monitoring, especially in multimodal and interdisciplinary treatment settings. Although results regarding baseline ctDNA levels are conflicting, pre-operative ctDNA appears to be predictive of pCR, as demonstrated in several retrospective or prospective cohort studies [68,69]. Combining ctDNA detection with an analysis of potentially detectable mutations could improve the prediction of recurrence and lead to substantial treatment adaptations [69].

Regarding baseline ctDNA levels, in a cohort of 36 patients, baseline ctDNA detection was a negative prognostic biomarker for OS [70]. The reduction in ctDNA levels during CRT limited the value of pre-operative ctDNA for prediction modeling.

In a cohort of 104 patients (29% pCR rate), preoperative ctDNA-positive rates were considerably lower in the ypCR than non-ypCR group, with a pathologic T stage (ypT) of 0–2 versus the ypT 3–4 group, and well-responding patients had a pathologic tumor regression grade of ypCAP 0–1 vs. the ypCAP 2–3 group [71]. Of note, pre-CRT ctDNA level was not correlated with response to treatment, as suggested in other studies [72,73]. However, all patients who experienced pCR had a negative pre-operative ctDNA, and a significant correlation was found between ctDNA detection and pCR (*p* = 0.02).

Post-hoc analysis of the prospective GEMCAD 1402 trial (180 patients) evaluated the addition of aflibercept to modified FOLFOX before CRT and subsequent surgery. ctDNA data were available for 72 patients; ctDNA was detectable in 83% of patients at baseline and only 15% of patients before surgery. No correlation was found between ctDNA and pCR, though ctDNA appeared as a prognostic variable for DFS and OS [74].

The monitoring of micro-RNAs (miRNAs) certainly has value in predicting response to NAT [75] and could be evaluated in both on tissue and blood samples. Several tissue miRNAs were associated with TRG, among which miR-630 and miR-622 reached sensitivities and specificities of 100% and were upregulated in responders [76]. miR-622 itself was further evaluated, but with conflicting results, as it appeared as highly expressed in pretreatment cells of non-responders (TRG4) [77]. Despite an unclear physiopathology of circulative miRNAs [78], several miRNAs have been identified as predictors of response after CRT. For example, miR-18b and miR-20a [79], as well as miR-125b [80] and miR-143 [81], were correlated with a response to CRT. To our knowledge, very few of these circulating miRNAs were externally validated. Wada et al. developed a blood-based model using a panel of eight different miRNAs, resulting in an AUC of 0.81 and a PPV of 51.4% for pCR prediction in an external cohort of 65 patients [82].

Single nucleotide polymorphisms (SNPs) are caused by a single nucleotide variation in the DNA sequence [83] and could have an impact on gene expression. Very few studies have evaluated the value of SNPs in LARC and in particular the response to treatment [75,84]. Nevertheless, promising results were shown with the role of rs61764370 SNP as a predictor of pCR, with increased 5-year PFS and OS when compared to the wild genotype patients [85]. In a cohort of 265 patients, five SNPS (rs744910, rs745103, rs6088619, rs10719, and rs17228212) had a predictive value for pCR.

Metabolic biomarkers, such as paraoxonase-1 (PON1) and α-ketoglutarate, could possibly be used for stratification between responders and non-responders or even pCR/non-pCR patients, as suggested by a single cohort study. Despite the small number of patients, this approach could be explored more thoroughly [86].

### 3.5. Pathomics

Analysis of anatomopathological images brings a new insight into prediction modeling. Using a cohort of 783 patients recruited from three different centers, Lou et al. developed a ResNet-18 network-based model [87]. On the external validation cohort (102 patients), the deep-learning model achieved an AUC of 0.72 (CI95% 0.59–0.84). The pathomics signature remained a significant predictor of pCR even after multivariate analysis. Of note, in the incorporated confounding features, only sex was a significant predictor (apart from the deep-learning model).

A combination of the radiomics and pathomics approaches was previously analyzed in a retrospective study conducted by Feng et al. [88]. With a training cohort of 303 patients, validation cohorts of 630 patients in total, and a prospective validation cohort of 100 patients, a radiopathomics model resulted in an AUC of 0.81 (CI95% 71.7–90.7%). Interestingly, the negative predictive value (NPV) reached 92.9% (86.2–99.5%), comforting clinicians in the case of a low predicted probability of pCR. Nevertheless, this high NPV should be put in balance with a pCR rate of 23%. This study remains one of the most well-conducted radiomics-based studies, with extensive validation and clinical impact, which could lead to treatment adaptations, such as dose escalation in non-pCR-predicted patients or NOM for pCR-predicted patients.

### 3.6. Microbiome

According to preclinical and clinical investigations, the gut microbiota may actively participate in various biochemical and pathophysiologic processes in the human body, including those involving inflammation and immunomodulation. However, data regarding response to CRT are particularly scarce in LARC patients. In a prospective longitudinal study, a decrease in LARC-related pathogens and an increase in *Lactobacillus* and *Streptococcus* was observed during CRT. Moreover, responders and non-responders exhibited differential microbiota, with *Coriobacteriaceae* and *Fusobacterium* being overrepresented in nonresponders. Combining ten biomarkers resulted in an AUC of 0.74 in a validation cohort (47 patients) [89]. Although the role of the microbiome on colorectal cancer tumorigenesis [90,91,92,93] was previously evaluated, the significance of these microbes regarding tumor response in LARC patients was unknown before the presented study. Given the latest data regarding the correlation between microbiota and tumor response in various localizations, the role of microbiota as predictors of response after CRT should definitely be investigated further. *Fusobacterium,* for instance, could be an indicator of tumor aggressiveness, as suggested by several studies [94,95].

## 4. Prediction of pCR Using Radiomics Model

A PubMed research query performed on the 14th of November 2022 using the terms ”Radiomics”, “Prediction”, “Complete Response”, and “Rectal Cancer” resulted in 61 studies published between 2016 and 2022, 37 of them having been published in 2021–2022, highlighting the large interest in this topic in the era of TNT and NOM. An overview of these studies with details on the type of NAT, population size, pCR rate, and existence of training/validation cohorts (external/internal) is further provided depending on the type of studied imaging modality.

Of the 61 identified studies, 13 studies were excluded for the following reasons: five were systematic, comprehensive, or narrative reviews; three were not radiomics-based studies; one had a primary endpoint of the prediction of MSI status; one focused on cervical cancer; one was a study protocol; one was a correction to the initial study; and one was a duplicate. As a result, 48 studies were further considered, among which the full article was available for 43 of them. Among the 48 studies, the vast majority (36/48: 75.0%) concerned MRI-only radiomics, and 4/48 (8.3%) and 8/48 (16.7%) concerned MRI-combined and non-MRI-based prediction models, respectively. Overall, a validation set was available for 30/48 (62.5%) of the studies, validation being prospectively conducted in only 2 studies (4.2%) [88,96]. The majority of prediction models were based on pre-CRT-only imaging (37/48: 77.1%), with a subset of models focusing on longitudinal (14.6%) and post-CRT (8.4%) imaging, when all studies were conducted in patients treated with CRT and not TNT.

Ranging from 16 to 592 patients, training cohort size did not significantly change when stratified by image modality (*p* = 0.81), timing of the imaging (*p* = 0.80), or when combining the image modality and the timing (*p* = 0.88).

As mentioned, only two trials had a prospective validation. Dinapoli et al. externally validated an MRI-based radiomics prediction model on two validation cohorts, one of which was prospectively conducted (THUNDER Trial). Despite having been prospectively included, analysis and validation were retrospectively performed. With a training cohort of 303 patients, 2 validation cohorts of respectively 480 and 150 patients, and a prospective validation cohort of 100 patients, the RAPIDS model presented by Feng et al. appears to be the only model to be fully validated. Combining baseline MRI radiomics features with pathomics nucleus and microenvironment features, the model achieved an AUC of 0.81 and a PPV of 0.51 in the prospective cohort. Combining the three sets of features (MRI, pathomics nucleus, and microenvironment features) significantly enhanced the model’s performance when compared to other single-modality models. Furthermore, in their effort of external validation, the authors developed a web-based platform that enables the widespread use of this model.

The low PPV values for most of the models must be put in balance with the low prevalence of pCR. When available with a range of 28.0–100.0, such non-invasive models show interesting results and should be further explored.

As mentioned, only 14.6% of the studies (7/48) used imaging with multiple time points (either pre- and mid-/post-CRT or pre- and during CRT). Although not significant, it should be acknowledged that the median training cohort size was smaller (35, CI95% 16–101.6) compared to the baseline (100.0, CI95% 86.0–130.8) and post-CRT (129.0, CI95% not available) cohorts only. The results of the longitudinally built radiomics models did not significantly outperform baseline prediction models in the validation cohorts. This could probably be explained by the low number of studies in which validation results were available in a longitudinal radiomics model (n = 5).

Delineation data were not retrieved for 4/48 studies, and most volumes were manually delineated (40/48–83.3%). When performed by multiple readers, multiple delineation was used as a feature selection step for only 12 of the studies (25.0%). Automatic or semi-automatic segmentation was used in four studies. The approach of Defeudis et al. should be highlighted because of the innovative pipeline it proposes. In addition to using manual segmentations defined by three different readers, an automatic segmentation was also evaluated. Interestingly, whereas the model using manual segmentation-based features performed highly on the training cohort (AUC 0.90), the model using the automatic segmentation features outperformed the other model on the validation cohort, with respective AUCs of 0.81 and 0.61 and accuracies of 75.0% and 65.0%.

These results highlight the need for broader external validation as well as quality assurance regarding the target definition, as further discussed in the limitations and perspectives paragraph.

Data regarding the 48 retrieved studies is hereby presented in Table 1, Table 2 and Table 3 for studies that focused on MRI-only, MRI-combined, and non-MRI imaging, respectively.

## 5. Limitations/Perspectives

Despite multiple studies and robust results, radiomics models fail to convince clinicians and are absent from current guidelines and clinical workflows.

The majority of models were tested on validation cohorts, despite only being prospectively evaluated.

Apart from a low level of evidence, several other concerns could explain the low acceptance of clinicians regarding radiomics-based models.

The majority of studies focused on GTV-extracted features, not taking into account variability in clinical target volumes (CTVs) and heterogeneity in the dosimetry planning and delivery. Given the complementary association between tumoral and peri-tumoral features, one might think that the definition of the CTV is important. Moreover, none of the studies evaluated the value of dose map-extracted features (dosiomics). Substantial differences in dose distribution can be observed depending on the treatment modality (3D-conformational, intensity-modulated RT, or volumetric arctherapy).

A positive impact was seen with automatically segmented GTVs [109], especially in the validation cohort. Such results highlight the need for quality assurance and possibly fully automatic workflows that would help standardize models’ development and validation and probably their generalization. In this aspect, the workflow proposed by Feng et al. would allow for such learning and application.

Previously presented models were mostly based on baseline MRIs, with several studies evaluating, with smaller samples, the value of multiple timepoint evaluations. Though this approach seems promising, the use of different imaging modalities provides new perspectives. For instance, the TEP ^64^Cu-ATSM-Rectum evaluates ^64^Cu-ATSM PET/CT as a new imaging biomarker in patients treated with CRT (NCT03951337).

Trans-omic analysis connects multi-omic layers and enables a better understanding of the physiopathology underlying, in our case, response to treatment. Though radiomics could be considered to be a macroscopic reflection of changes happening at the microscopic level, trans-omics could connect the information provided by genomics, transcriptomics, and proteomics using computational data. A complete understanding of the mechanisms of response to neo-adjuvant therapy would not only allow for prediction, but most likely for patient’s selection before treatment is even performed. However, to this day, and despite several teams working on this matter, no studies focusing on the prediction of pCR using a trans-omics approach have been published.

Finally, the recent change in LARC management and the advent of TNT limit the validity of previously presented studies, as none was performed or evaluated in TNT-treated patients. This lack of data is explained by the current workflow that most radiomics studies use. Whereas genomic data are often acquired prospectively, radiomics studies mostly consist of post-hoc retrospective analyses. Given the impact of TNT on pCR rates, one can expect the prediction from previous models to be inaccurate. New radiomics models must thus be developed. Promising results regarding the use of neoadjuvant immune checkpoint inhibitors (ICIs) could revolutionize the management of LARC patients. Though the addition of pembrolizumab to TNT did not modify the pCR rate in unselected LARCs patients [143], the use of nivolumab plus ipilimumab seems to be very promising in dMMR (mismatch repair-deficient) tumors. Combining PD-1 and CTLA-4 blockage led to a 60% pCR rate in dMMR patients, and no patients with a pMMR tumor experienced a pCR [144].

## 6. Conclusions

In recent years, neoadjuvant therapy for locally advanced rectal cancer has seen tremendous modifications. Neoadjuvant CRT in combination with pre-operative chemotherapy improves LRFS and pCR rates, opening the possibility of non-operative management and organ preservation without compromising overall survival. Given the challenge of pCR prediction, multiple approaches have been developed, and radiomics-based models especially have achieved promising results but lack external validation. Substantial efforts have been made regarding the quality of published radiomics models. Such approaches could be combined and/or compared to other promising approaches, such as circulating DNA.

## Figures and Tables

**Table 1:** Summary of MRI-only-based radiomics prediction model.

Study Year	Studied Modality of Imaging Timing	External Validation - Prospective Validation	Timing for Data Acquisition - Timing of Analysis: Prospective/Retrospective	Cohort Size	Delineation	Assessment of Robustness	Extraction Software - Statistical Approach	Predicted Outcome	Correction for Site Heterogeneity	Type of NAT: CRT vs. TNT	pCR Rate	AUC	Accuracy	PPV	AUC	Accuracy	PPV
												Training			Validation		
Antunes et al. [97] 2020	MRI: T2 baseline	Y - N	Retrospective - Retrospective	Training: 60, validation: 44	Manual: single reader	Y	In-house - Random forest	pCR	N	CRT	Training: 21.7%, validation: 22.7%	0.70	70.5% for the overall cohort	N/A	0.71	N/A	N/A
Boldrini et al. [98] 2019	MRI: T2/T1S longitudinal	N-4 cross fold validation - N	Retrospective - Retrospective	16	Manual-Cooperation of 2 radiation oncologists	N	Moddicom (R) - Wilcoxon–Mann–Whitney	pCR	N/A	CRT	12.5%	0.93	N/A	N/A	N/A	N/A	N/A
Boldrini et al. [99] 2022	MRI: T2 baseline	Y - N	Retrospective - Retrospective	Training: 162 (see Dinapoli et al., 2018); testing: 59	Manual: single reader, with review by an independent reader	N	Moddicom (R) - GLM	pCR	N	Training: see Dinapoli et al., 2018; validation: CapOX followed by CRT	Training 28%; validation cohort: 16.9%	0.73	N/A	N/A	0.83	65.0	28.0
Bulens et al. [100] 2020	MRI: T2/DWI/ADC baseline	Y - N	Retrospective - Retrospective	Training: 70, validation: 55	Manual consensus of 1 radiation oncologist and 1 radiologist	N	In-house - LASSO logistic regression model	pCR	N	CRT	Training: 17%, validation: 15%	0.86	82.0	77.0	0.87	84.0	100.0
Chen et al. [101] 2022	MRI: T2 baseline	Y - N	Prospective - Retrospective	Training: 91, validation: 46	N/A	N	PyRadiomics - Logistic regression	pCR	N	CRT	Training: 16.5%, validation: 19.5% *	0.96	N/A	N/A	0.87	N/A	N/A
Chen et al. [102] 2020	MRI: T2 longitudinal (pre/post)	Y - N	Retrospective - Retrospective	Training: 26, validation: 13	Manual: single reader	N/A	IBEX - SVM	pCR	N/A	CRT	25.6%	N/A	96.2%	N/A	N/A because of missing data	N/A	N/A
Cheng et al. [103] 2021	MRI: T2/T2FS/T1 baseline	Y - N	Retrospective - Retrospective	Training = 128, validation: 65	Manual: single reader (+ inter-reader variability assessment on 30 patients)	Y	PyRadiomics - Logistic regression	pCR	N/A	CRT	Training: 15.6%, validation: 16.9%	0.96	93.8	N/A	0.91	84.6	N/A
Chiloiro et al. ** [104] 2022	MRI: N/A post-CRT	N - N	Retrospective - Retrospective	144	N/A	N/A	N/A - N/A	pCR	N/A	CRT	N/A	0.84	N/A	N/A	N/A	N/A	N/A
Coppola et al. [105] 2021	MRI: T2/DWI baseline	N - N	Retrospective - Retrospective	40	Manual: Single reader	N/A	N/A - Random forest	near pCR + pCR	N/A	CRT	37.5% *	0.90	N/A	80.0	N/A	N/A	N/A
Cui et al. [106] 2018	MRI: T2/T1/ADC Baseline	Y - N	Retrospective - Retrospective	Training: 131; validation: 55	Manual	N/A	Analysis kit-GE - Logistic regression	pCR	N/A	CRT	Training: 16.8%, validation: 16.4%	0.95	87.8	N/A	0.97	94.5	N/A
Cusumano et al. [107] 2021	MRI (low field): T2/T1S longitudinal	Y - N	Retrospective - Retrospective	Training: 16, validation: 43	Manual-cooperation of 2 radiation oncologists	N	Moddicom (R) - Multiple logistic regression	pCR	N/A	CRT	Training: 12.5%, validation: 27%	0.93	N/A	N/A	N/A	77.0	N/A
Cusumano et al. [108] 2021	MRI: T2? baseline	N-internal cross validation - N	Retrospective - Retrospective: 2 institutions	195 (institution1: 136; institution2: 59)	Manual: nb readers?	N/A	Moddicom (R) - Multiple logistic regression	pCR	N	CRT	Institution1: 22%; institution 2: 25%	0.72 (after cross validation)	N/A	N/A	N/A	N/A	N/A
Defeudis et al. [109] 2022	MRI: T2/ADC baseline	Y - N	Retrospective - Retrospective	Training: 47, testing: 20, validation: 28	Manual: 3 readers + assessment by 2 supplementary readers	N	PyRadiomics - SVM	pCR	N	CRT	16.8%	0.90	83.0	74.0	0.61	68.0	75.0
					Automatic segmentation	N	PyRadiomics - SVM	pCR	N	CRT	16.8%	0.86	78.0	71.0	0.81	75.0	75.0
Delli Pizzi et al. [110] 2021	MRI: T2 baseline	N - N	Retrospective - Retrospective	72	Manual: 2 readers	Y	PyRadiomics - Partial least-squares regression	near pCR + pCR	N/A	CRT	47.2%*	0.79	N/A	N/A	N/A	N/A	N/A
Dinapoli et al. [96] 2018	MRI: T2 baseline	Y - N	Training: retrospective, validation 1: retrospective, validation 2: prospective - Retrospective	Training: 162, validation cohort 1: 34, validation cohort 2: 25	Manual-training: cooperation of 2 radiation oncologists + 2 radiologists, validation 1: cooperation of 2 different radiation oncologists and radiologists, validation 2: same as training	N	Moddicom (R) - GLM	pCR	N/A	CRT	Training 28%; validation cohort 1: 21%, validation cohort 2: 28%	0.73	N/A	N/A	Cohort 1: 0.75, cohort 2: 0.79	N/A	N/A
Fu et al. [111] 2020	MRI: DWI baseline	N-internal cross validation - N	Retrospective - Retrospective	43	Manual: single reader	N/A	PyRadiomics - Neural network	near pCR + pCR	N	CRT	51.2%	0.73 (after cross validation)	N/A	N/A	N/A	N/A	N/A
Van Griethuysen et al. [112] 2020	MRI: T2/DWI/ADC baseline	Y - N	Retrospective - Retrospective	Training: 86, validation: 47	Semi-automatic + manual refinement	N	PyRadiomics - Logistic regression	pCR	N	CRT	Training: 20.9%, validation: 21.3%	0.71	N/A	N/A	0.77	N/A	N/A
Horvat et al. [113] 2018	MRI: T2 post-CRT	N - N	Retrospective - Retrospective	114	Manual: consensus of 2 readers	N/A	In-house - Random forest	pCR	N	CRT	18%	0.93	N/A	72.0%	N/A	N/A	N/A
Horvat et al. ** [114] 2022	MRI: T2 baseline	Y - N	Retrospective - Retrospective	Training: 114? validation: 50	Manual: 2 readers	N/A	N/A - N/A	pCR	N/A	CRT	N/A	N/A	N/A	N/A	0.83	N/A	57.0
Jayaprakasam et al. ** [115] 2021	MRI: T2 baseline	N-internal cross validation - N	Retrospective - Retrospective	236	Manual: 3 readers	N/A	CERR - SVM	pCR	N/A	CRT	N/A	0.89	83.9	52.5	N/A	N/A	N/A
Li et al. [116] 2019	MRI: T2 longitudinal (pre/post)	Y - N	Retrospective - Retrospective	Training: 87, validation: 44	Manual: single reader, with review by an independent reader	N	IBEX - LASSO logistic regression model	pCR	N/A	CRT	Training: 20.7%, validation: 20.5%	0.92	N/A	N/A	0.87	N/A	N/A
Liu et al. [117] 2017	MRI: T2/DWI baseline	Y - N	Retrospective - Retrospective	Training: 16, validation: 43	Manual: 2 readers	Y	MATLAB workpackage - SVM	pCR	N/A	CRT	Training: 17.1%, validation: 17.1%	0.97	96.1	91.7	0.98	94.3	90.0
Mbanu et al. [118] 2022	MRI: T2 baseline	Y - N	Retrospective - Retrospective	Training: 200, validation: 104	Manual: 2 readers	Y	PyRadiomics - Logistic regression	cCR	N	CRT	50% (selected patients for matching cohorts)	0.76	N/A	N/A	0.68	N/A	N/A
Nardone et al. [119] 2022	MRI: T2/DWI/ADC longitudinal (pre/post)	Y - N	Retrospective - Retrospective	Training: 37, 2 validation cohorts: 33 + 30	Manual: 2 readers + assessment by 2 supplementary readers	Y	Life X- Logistic regression	pCR	N	CRT	Training: 27%; validation cohort 1: 18%, validation cohort 2: 17%	0.87	73.0	50.0	Cohort 1: 0.92, cohort 2: 0.88	Cohort 1: 72.7, cohort 2: 80.0	Cohort 1: 40.0, cohort 2: 44.4
Nie et al. [120] 2016	MRI: T2/T1/DWI/DCE Baseline	N-4 cross fold validation - N	Retrospective - Retrospective	48	Manual: single reader	N/A	N/A - Neural network	pCR	N/A	CRT	23%	0.89	N/A	N/A	N/A	N/A	N/A
Pang et al. [121] 2021	MRI: T2 post-CRT	Y - N	Retrospective - Retrospective	Training: 107, internal validation: 46, external validation: 34	Automatic segmentation	N/A	PyRadiomics - SVM	pCR	N	CRT	Training: 33.6%, internal validation: 17.4%, external validation: 17.6%	0.92	86.0	N/A	Internal validation: 0.83, external validation: 0.82	Internal validation: 80.4, external validation: 85.3	N/A
Peterson et al. ** [122] 2022	MRI: N/A baseline	N - N	Retrospective - Retrospective	131	N/A	N/A	N/A - Machine learning?	pCR	N/A	CRT	26.7%	0.73	N/A	N/A	N/A	N/A	N/A
Petkovska et al. [123] 2020	MRI: T2 baseline	Y (sub-set of patients delineated by different readers) - N	Retrospective - Retrospective	Training: 102, validation: 66 (out of the training cohort but with different delineations)	Manual: 1 reader (+ 2 readers for validation)	N	CERR - SVM	pCR	N	CRT	19%	0.75	N/A	N/A	Reader 1: 0.75, reader 2: 0.71	N/A	N/A
Shaish et al. [124] 2020	MRI: T2 baseline	N-internal cross validation - N	Retrospective - Retrospective	Training: 112, validation: 23	Manual: single reader	Y	PyRadiomics - Logistic regression	pCR	N	CRT	15%	0.80	N/A	N/A	N/A	N/A	N/A
Shi et al. [125] 2019	MRI: T2/T1/ADC/DCE longitudinal (pre/mid)	N-internal cross validation - N	Retrospective - Retrospective	35	Manual	N	N/A - Neural network	pCR	N/A	CRT	22.2%	0.86	N/A	N/A	N/A	N/A	N/A
Shin et al. [126] 2022	MRI: T2/ADC post-CRT	Y - N	Retrospective - Retrospective	Training: 592, validation: 306	Manual: single-reader assessment by 1 supplementary reader (+ inter-reader variability assessment on 40 patients)	Y	PyRadiomics - LASSO logistic regression model	pCR	N	CRT	Training: 19.3%, validation: 24.5%	0.89	N/A	N/A	0.82	N/A	46.3
Song et al. [127] 2022	MRI: T2 baseline	Y - N	Retrospective - Retrospective	674 patients	Manual: 4 readers and 2-step validation	N	N/A - SVM	pCR	N	CRT	25.8%	0.99	96.4	96.1	0.79	93.3	94.0
Tang et al. [128] 2022	MRI: T2 baseline	Y - N	Training: see Dinapoli et al. 2018; validation: retrospective - Training: see Dinapoli et al., 2018; validation: retrospective	Training: see Dinapoli et al., 2018; validation: 88	Manual: single reader	N/A	Moddicom (R) - Logistic regression	pCR	N	CRT	Training: see Dinapoli et al., 2018; validation: 13.6%	0.93	N/A	N/A	0.93	N/A	N/A
Wan et al. [129] 2020	MRI: T2/DWI longitudinal (pre/post)	Y - N	Retrospective - Retrospective	Training: 116, validation: 49	Manual: single-reader assessment by 1 supplementary reader	N/A	Radcloud - Logistic regression	pCR	N/A	CRT	Training: 16.4%, validation: 16.3%	0.91	N/A	N/A	0.91	N/A	N/A
Wei et al. ** [130] 2022	MRI: N/A baseline	Y - N	Retrospective - Retrospective	Training: 100, validation: 51	Manual	N/A	N/A - Random forest	pCR	N/A	CRT	N/A	0.91	76.0	N/A	0.87	77.3	N/A
Yi et al. [131] 2019	MRI: T2 baseline	Y - N	Retrospective - Retrospective	Training: 93, validation: 40	Manual: 2 readers	Y	MaZda - SVM	pCR	N	CRT	23.9%	0.91	N/A	N/A	0.87	N/A	N/A

Abbreviations: *: near pCR when indicated, **: full text not available, nb: number, NAT: neoadjuvant treatment, CRT: chemoradiotherapy, TNT: total neoadjuvant treatment, pCR: pathological complete response, AUC: area under the curve, PPV: positive predictive value, N/A: not available, Y: yes, N: no, MRI: magnetic resonance imaging, DWI: diffusion-weighted imaging, DCE: dynamic contrast enhanced, ADC: Apparent Diffusion Coefficient, T1S: T1-Star, T2FS: T2 fat saturated, SVM: support vector machine, LASSO: least absolute shrinkage and selection operator, GLM: generalized linear model.

**Table 2:** Summary of MRI-combined-based radiomics prediction model.

Study Year	Studied Modality of Imaging Timing	External Validation - Prospective Validation	Timing for Data Acquisition - Timing of Analysis: Prospective/Retrospective	Cohort Size	Delineation: Manual/Auto, nb Reader	Assessment of Robustness	Extraction Software - Statistical Approach	Predicted Outcome	Correction for Site Heterogeneity	Type of NAT: CRT vs. TNT	pCR Rate	AUC	Accuracy	PPV	AUC	Accuracy	PPV
												Training			Validation		
Bordron et al. [132] 2022	MRI: T2/DWI CE-CT baseline	Y - N	Retrospective - Retrospective	Training: 64, testing: 60	Manual: single reader (+ inter-reader variability assessment on 25 patients)	Y	MIRAS - Neural network: multilayer perceptron	pCR	Y (Combat)	CRT	Training: 14%, validation: 8%	0.95	90.0	45.0	0.81	85.5	44.4
Capelli et al. [133] 2022	MRI: T2/ADC PET baseline	N/A - N	Retrospective - Retrospective	50	Manual:consensus of 2 radiation oncologists	N/A	PMOD - Logistic regression	pCR	N/A	CRT	50	0.86	74.0	N/A	N/A	N/A	N/A
Feng et al. [88] 2022	MRI: T2/T1/DWI pathological slides baseline	Y - Y	Retrospective: training + validation + prospective validation - Retrospective: training + validation + prospective validation	Training: 303, validation 1 + 2: 480 + 150, prospective: 100	Manual	N/A	PyRadiomics - Neural network: VGG-19	pCR	N	CRT	Training: 28%, validation 1/2: 22%/20%, prospective: 23%	0.87	N/A	0.64	Validation 1: 0.86, validation 2: 0.87, prospective: 0.81	N/A	Validation 1: 0.50, validation 2: 0.47, prospective: 0.51
Giannini et al. [134] 2019	MRI: T2/DWI PET baseline	N - N	Prospective - Retrospective	52	Semi-automatic	N/A	MATLAB workpackage - Logistic regression	near pCR + pCR	N/A	CRT	42.3%	0.86	N/A	N/A	N/A	N/A	N/A

Abbreviations: nb: number, NAT: neoadjuvant treatment, CRT: chemoradiotherapy, TNT: total neoadjuvant treatment, pCR: pathological complete response, AUC: area under the curve, PPV: positive predictive value, N/A: not available, Y: yes, N: no, MRI: magnetic resonance imaging, DWI: diffusion-weighted imaging, CE: contrast enhanced, CT: computed tomography, ADC: apparent diffusion coefficient, PET: positron emission tomography.

**Table 3:** Summary of non-MRI-based radiomics prediction model.

Study Year	Studied Modality of Imaging Timing	External Validation - Prospective Validation	Timing for Data Acquisition - Timing of Analysis: Prospective/Retrospective	Cohort Size	Delineation: Manual/Auto, nb Reader	Assessment of robustness	Extraction Software - Statistical Approach	Predicted Outcome	Correction for Site Heterogeneity	Type of NAT: CRT vs. TNT	pCR Rate	AUC	Accuracy	PPV	AUC	Accuracy	PPV
												Training			Validation		
Bibault et al. [135] 2018	NonCE-CT baseline	N-internal cross validation - N	Retrospective - Retrospective	95	Manual: 2 readers	N	IBEX - Neural network: tensor flow	pCR	N	CRT	23.1%	NaN	NaN	NaN	0.72	80.0	68.0
Hamerla et al. [136] 2019	NonCE-CT baseline	N-internal cross validation - N	Retrospective - Retrospective	169	Manual: consensus of 2 radiation oncologists	N	PyRadiomics - Random forest	pCR	N/A	CRT	13%	NaN	87.0 (before correction for imbalanced data); 50.0 (after correction for imbalanced data)	NaN	NaN	NaN	NaN
Lovinfosse et al. [137] 2017	PET baseline	N - N	Retrospective - Retrospective	86	Semi-automatic	N/A	In-house - Logistic regression	pCR	N/A	CRT	9.1%	NaN	NaN	NaN	NaN	NaN	NaN
Lutsyk et al. [138] 2021	NonCE-CT baseline	Y - N	Retrospective - Retrospective	Training: 98, validation: 42	Manual	N	PyRadiomics - Neural network: multilayer perceptron	pCR	N	CRT	27.1%	0.87	98.0	98.6	NaN	67.0	77.0
Mao et al. [139] 2022	NonCE-CT baseline	Y - N	Retrospective - Retrospective	Training: 151, validation: 65	Manual: 2 readers + assessment by 2 supplementary readers	Y	MaZda - Logistic regression	pCR	N/A	CRT	Training: 19.9%, validation: 21.5%	0.93	87.0	60.5	0.87	86.0	70.6
Shen et al. [140] 2020	PET baseline	N-internal cross validation - N	Retrospective - Retrospective	169	Manual	N	In-house - Random forest	pCR	N/A	CRT	13%	0.87–0.97 (before cross validation)	89.0 (after cross validation)	67.0 (after cross validation)	NaN	NaN	NaN
Yuan et al. [141] 2020	NonCE-CT baseline	Y - N	Retrospective - Retrospective	Training = 60, validation: 31	Manual	N	In-house - Random forest > SVM	pCR	N	CRT	Training: 23.4%, validation: 19.4%	NaN	NaN	NaN	NaN	83.9	NaN
Zhuang et al. [142] 2021	CE-CT baseline	Y - N	Training + validation: post-hoc analysis of the FORWAC trial - Retrospective	Training: 113, validation: 64	Manual: 2 readers	Y	PyRadiomics - GLM	pCR	N	FORWAC trial: 3 arms: arm A: LV5FU2 + RT, arm B: FOLFOX + RT, arm C: FOLFOX without RT	Training: 17.7%, validation: 17.2%	1.00	0.97	NaN	0.82	81.0	NaN

Abbreviations: nb: number, NAT: neoadjuvant treatment, CRT: chemoradiotherapy, TNT: total neoadjuvant treatment, pCR: pathological complete response, AUC: area under the curve, PPV: positive predictive value, N/A: not available, Y: yes, N: no, MRI: magnetic resonance imaging, CE: contrast enhanced, CT: computed tomography, PET: positron emission tomography, GLM: generalized linear model.
